# A Detection Method of Novel Class for Radiation Source Individuals Based on Feature Distribution and Isolation Forest

**DOI:** 10.3390/s25185747

**Published:** 2025-09-15

**Authors:** Qiang Pan, Lei Shi, Changzhao Feng, Yinan Li, Congcong Wang, Yuefan Du, Zhiyi Chen

**Affiliations:** 1 School of Aerospace Science and Technology, Xidian University, Xi’an 710126, China; 22131214221@stu.xidian.edu.cn (Q.P.); 22131214191@stu.xidian.edu.cn (C.F.); yuefandu@stu.xidian.edu.cn (Y.D.); 2 The China Academy of Space Technology, Xi’an 710100, China; liyinan_cast@126.com (Y.L.); sunseacc@163.com (C.W.); 3 Xinjiang Yilin Industry Share Co., Ltd., Urumqi 830000, China; yilings@126.com

**Keywords:** specific emitter identification, novel-class detection, isolated forest, anomaly discrimination, ADS-B signal

## Abstract

Traditional specific emitter identification (SEI) systems often suffer significant performance degradation when confronted with previously unseen signal sources, underscoring the critical need for accurate detection and rejection of novel-class instances. To address this limitation, we propose an Integrated Deep Feature Representation and Isolation Forest (IDFIF) method for identifying novel-class radiation emitters. IDFIF begins by employing a convolutional neural network (CNN) to extract embedding features from raw In-phase/Quadrature (IQ) signals, enhancing inter-class separability while suppressing intra-class variability. These deep features are then used to construct an unsupervised iForest that learns the statistical distribution of known classes, enabling the effective detection of anomalies via a threshold-based scoring mechanism. Experiments conducted on a real-world ADS-B dataset demonstrate that the proposed method achieves a novel-class detection accuracy of over 94%, significantly outperforming comparative methods. Furthermore, the method exhibits low sensitivity to known-class samples, thereby ensuring robustness and generalization under open-set conditions. The proposed IDFIF method is promising for deployment in challenging electromagnetic environments.

## 1. Introduction

In increasingly complex electromagnetic environments, Specific Emitter Identification (SEI) has become a critical component of electronic reconnaissance and countermeasure systems [1,2]. By extracting unique radio “fingerprints” embedded in emitter signals, SEI enables the precise identification of emitter types, states, and even individual devices. This capability plays a critical role in characterizing adversarial electronic assets and informing strategic situational awareness [3,4].

However, the increasing complexity of electromagnetic environments, along with the rapid proliferation of modern communication devices, poses significant challenges for conventional signal recognition systems. These evolving conditions highlight a fundamental limitation of existing SEI models, their inherent inability to generalize beyond the closed-set of predefined training classes. This limitation motivates the development of more adaptive and robust recognition frameworks capable of functioning under open-set conditions. In scenarios involving novel-class radiation source individuals, conventional closed-set recognition models often experience severe performance degradation, leading to frequent misclassifications or missed detections that undermine recognition accuracy [5]. Therefore, enhancing recognition systems with the ability to detect and reject unknown threats, particularly novel-class emitters, has become a central challenge in the development of SEI systems [6,7].

Addressing this challenge requires recognition systems to determine whether incoming signal samples belong to known classes or originate from previously unseen novel classes [8,9]. In practical scenarios, signal data typically exhibits substantial intra-class variation and limited inter-class separability, primarily due to channel interference, receiver noise, and other environmental distortions. These characteristics severely hinder the effectiveness of conventional recognition approaches in detecting novel-class instances. To overcome these limitations, this paper proposes IDFIF, which integrates deep embedding extraction with unsupervised anomaly detection to effectively distinguish between known-class and novel-class signal samples without requiring any prior exposure to unseen categories.

To situate the Novel-Class Radiation Source Individual Detection (NCSD) task within the broader landscape of machine learning, we briefly review representative approaches developed in other domains, such as facial and voice recognition. In these domains, detecting novel-class instances is likewise regarded as a critical research challenge [10,11]. Within the context of NCSD, prior studies have explored traditional closed-set classification, binary classification, and open-set recognition (OSR) paradigms as potential solutions for novel-class detection. However, traditional classifiers, limited by closed-set assumptions and static model structures, fail to distinguish novel-class instances, thereby reducing the responsiveness of electronic countermeasure systems. As a result, novel-class detection has been formulated as a binary classification problem (known vs. novel-classes), where positive and negative samples correspond to novel and known classes, respectively [12]. A commonly adopted strategy involves training classifiers under supervised or semi-supervised settings, often using a combination of real measurements and synthetically generated pseudo-samples. However, obtaining labeled data from novel classes in electromagnetic environments is often impractical [13], thereby limiting the applicability of these methods in NCSD scenarios. Consequently, researchers have explored unsupervised anomaly detection techniques, such as Liu et al.’s iForest [14], Yang’s KNN-based novelty detection [15,16], Rettig’s online anomaly detection for streaming data [17,18], and Grandvalet’s SVM-based rejection method [19]. Although these methods eliminate the need for labeled novel-class samples, their detection performance heavily relies on the assumption of clear feature separation between known and novel classes. In practice, however, highly overlapping feature distributions in real-world electromagnetic channels significantly undermine the ability of anomaly detection models to maintain low false-alarm rates.

Recently, increasing attention has been devoted to open-set recognition (OSR) [20], which aims to classify known classes while accurately rejecting unknown inputs. Bendale and Boult [21] proposed OpenMax, the first deep neural network–based framework explicitly designed for open-set conditions. Rozsa et al. [22] investigated the adversarial robustness of deep neural networks utilizing softmax and OpenMax layers. Their findings indicate that while OpenMax reduces vulnerability to conventional adversarial attacks, it remains susceptible to more sophisticated techniques targeting the learned feature representations. Although OSR techniques have shown promise in open-world classification tasks, they often rely on large-scale labeled datasets and struggle to maintain robustness when applied to radiation source signals characterized by significant intra-class variation and inter-class overlap [23,24].

In summary, existing methods for novel-class detection in electromagnetic environments face two fundamental challenges:

1. Radiation source signal features exhibit significant overlap, with substantial intra-class variability and limited inter-class separability, which complicates the modeling of accurate class boundaries.
2. The absence of robust detection mechanisms within current SEI systems often results in missed or incorrect identification of novel-class and impairing electronic situational awareness.

To overcome these challenges, this paper proposes the IDFIF. The proposed method constructs a detection framework trained exclusively on known-class data, thereby eliminating the need for prior exposure to novel classes. Given the substantial intra-class variability and inter-class overlap in radiation source signals, the method employs a deep CNN to explicitly enhance feature space separability and improve robustness against channel distortions, which are critical for reliable novel-class detection. The resulting embeddings are then processed by an iForest to identify outliers based on statistical deviations in the feature space. A threshold-based rejection mechanism is subsequently employed to enhance detection reliability by minimizing false positives. Compared to conventional classifiers and binary detectors, the proposed method offers a scalable and data-efficient solution for the NCSD task.

The remainder of this paper is organized as follows: Section 1 introduces the concept of radiation source individual recognition and outlines the key challenges currently faced in this domain. Section 2 presents the dataset and analyzes the underlying feature distributions. Section 3 details the algorithmic framework and implementation of the proposed IDFIF. Section 4 outlines the experimental setup, evaluates the performance, and discusses key findings. Finally, Section 5 summarizes the main contributions and provides concluding remarks.

## 2. Inter-Class and Intra-Class Variation in Radiation Source Individuals

### 2.1. Dataset Overview of the Radiation Source Individuals

To facilitate the study of individual radiation source recognition, we utilize a real-world dataset based on the Automatic Dependent Surveillance Broadcast (ADS-B) system. ADS-B is a standardized aviation surveillance protocol in which onboard transmitters periodically broadcast flight parameters such as position, altitude, and velocity over a dedicated 1090 MHz carrier frequency. Despite the standardization, differences at the device level in circuitry, manufacturing precision, and operational states introduce subtle but distinctive variations in signal characteristics across individual transmitters. These characteristics make ADS-B a valuable platform for SEI. In the context of novel-class detection, the structured format and large number of emitters in the ADS-B system provide ideal conditions for evaluating the generalization capability of detection models. In this study, we employ the publicly available ADS-B dataset collected by Ya Tu et al. [25] at Harbin Institute of Technology. The dataset was based on the Mode S data link under the 1090ES standard, and signal acquisition was conducted using the SM200B software-defined radio platform.

**Figure 1:**
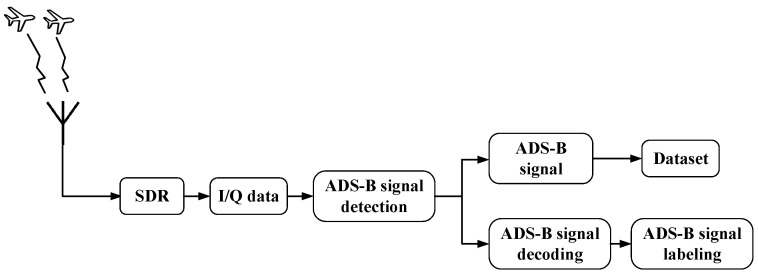
Overall process of the dataset collection system.

Each ADS-B message contains an 8 μs synchronization preamble followed by a data block encoded using pulse position modulation. Two message formats are adopted, including a 56-bit short format and a 112-bit long format. Both formats include the International Civil Aviation Organization address, which uniquely identifies each aircraft. The signal acquisition system, illustrated in Figure 1, consists of an SM200B SDR receiver, a high-performance computing terminal with decoding software, an omnidirectional antenna operating at 1090 MHz, and an open environmental setup. Through continuous collection and automated labeling, the dataset comprises a total of 26,613 long-format IQ signals from 1661 aircraft types and 167,234 short-format signals from 1713 aircraft types. This large-scale and well-structured dataset provides a robust empirical foundation for evaluating novel-class detection methods.

### 2.2. Time-Domain Observation and Physical-Layer Analysis

A preliminary analysis was conducted to examine the physical-layer properties of ADS-B signals. In particular, signal variation patterns were examined in the time domain to assess waveform consistency and individual-level differences.

**Figure 2:**
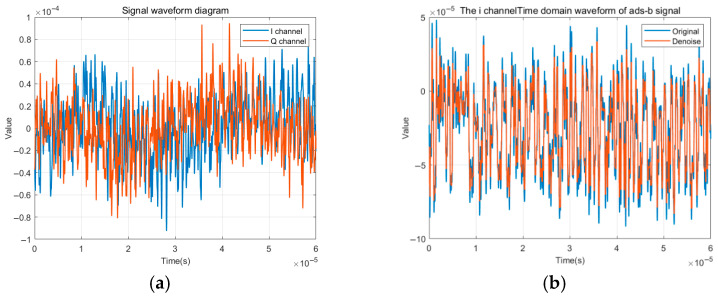
(**a**) Original waveform diagram of an individual ADS-B radiation source; (**b**) time-domain waveform before and after signal wavelet denoising.

As shown in Figure 2a, In-phase signals from several emitters were visualized to investigate similarities and differences at the class level. Observable fluctuations were evident even among samples from the same emitter, reflecting intra-class variability. Subtle structural differences were also observed across emitters, suggesting the presence of latent individual-specific characteristics embedded in the signal morphology. It is important to note that ADS-B signals are modulated using In-phase and Quadrature components, which are inherently 90 degrees out of phase. Due to the high redundancy between these channels and the need for computational efficiency, only the In-phase component was retained in the subsequent analysis and modeling.

Wavelet-based denoising was applied as a diagnostic tool to improve the interpretability of signal features. Specifically, the Daubechies-1 wavelet was used for multi-scale decomposition, along with thresholding to suppress high-frequency noise. As shown in Figure 2b, this process smoothed the waveform while preserving key modulation boundaries and localized transitions. Although not included in the actual feature extraction pipeline, wavelet denoising improves visual clarity and supports the understanding of intra-class variation and channel noise interference.

These observations confirm that radiation source signals exhibit significant variability at the physical layer. This poses challenges for boundary modeling and highlights the need for robust feature extraction methods such as CNNs in the proposed method.

### 2.3. Feature Space Visualization and Distribution Analysis

**Figure 3:**
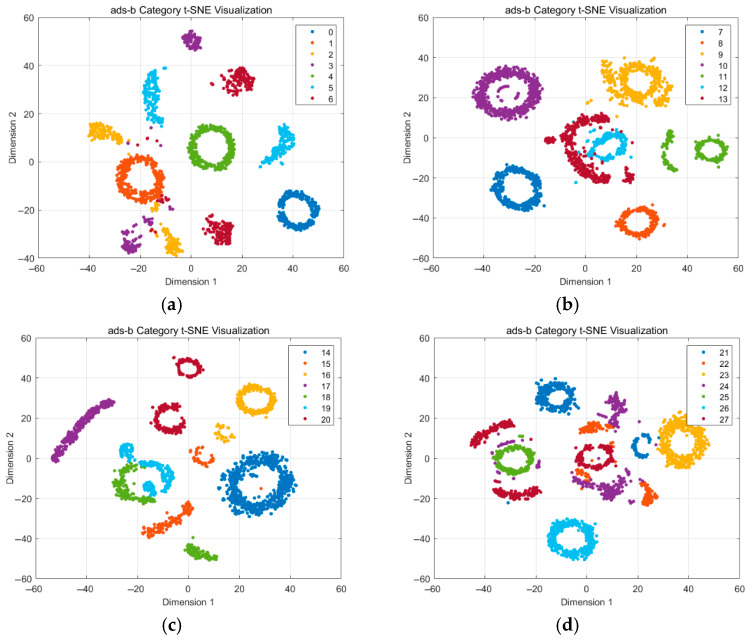

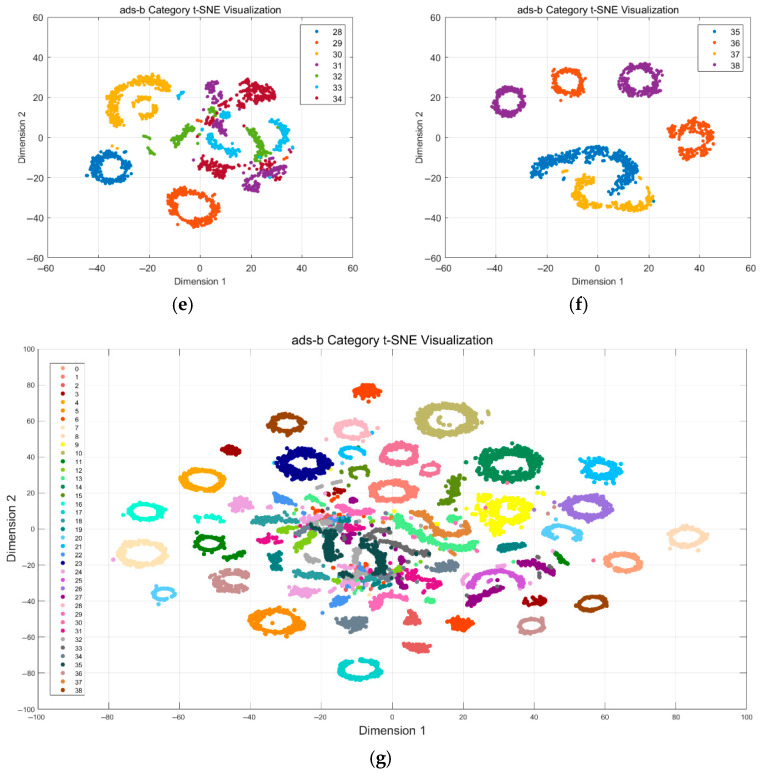
t-SNE visualizations of CNN feature embeddings for ADS-B signal categories. (**a**) Class 0–6; (**b**) class 7–13; (**c**) class 14–20; (**d**) class 21–27; (**e**) class 28–34; (**f**) class 35–38; (**g**) class0–38.

Following the physical-layer signal analysis, we investigated the distributional characteristics of radiation source signals in the learned feature space. The goal was to assess intra-class and inter-class variation patterns that directly affect classification and detection performance. To this end, a CNN feature extractor is trained on the short-format ADS-B signals to obtain compact embedding representations. The embeddings visualized in this section were extracted from the final fully connected layer of the trained CNN, prior to softmax activation. These representations are the same feature vectors used as input to the iForest anomaly detection module in the proposed IDFIF framework. The first 39 classes (Class 0–38) were selected from the dataset, and the t-Distributed Stochastic Neighbor Embedding (t-SNE) was applied for visualization [26]. To facilitate detailed observation, the 39 classes are divided into six groups, and the corresponding t-SNE plots are shown in Figure 3. Additionally, to provide a global perspective of the feature space structure, a full t-SNE plot visualizing all 39 classes simultaneously is presented as Figure 3g. Although visually dense, this plot helps reveal the macroscopic distribution patterns and the general clustering behavior across all categories.

The visualizations reveal a number of critical structural patterns. First, within each subfigure, samples from the same class often exhibit considerable spread, suggesting high intra-class variability. Second, several class pairs show overlapping clusters or indistinct boundaries, indicating insufficient inter-class separability. Notable examples include overlaps between: class 2 and some samples of class 3 and class 1 in Figure 3a; class 12 and class 13 in Figure 3b; class 18 and class 19 in Figure 3c; class 26 and some samples of class 27 in Figure 3d; some samples of class 27 and class 22 as well as class 24 in Figure 3d; class 31 and class 32, class 33 and class 34 in Figure 3e; class 35 and class 37 in Figure 3f. These observations confirm that certain classes exhibit localized clustering, the overall feature space was characterized by considerable intra-class variation and small inter-class separability. This distributional pattern complicates reliable boundary modeling.

**Figure 4:**
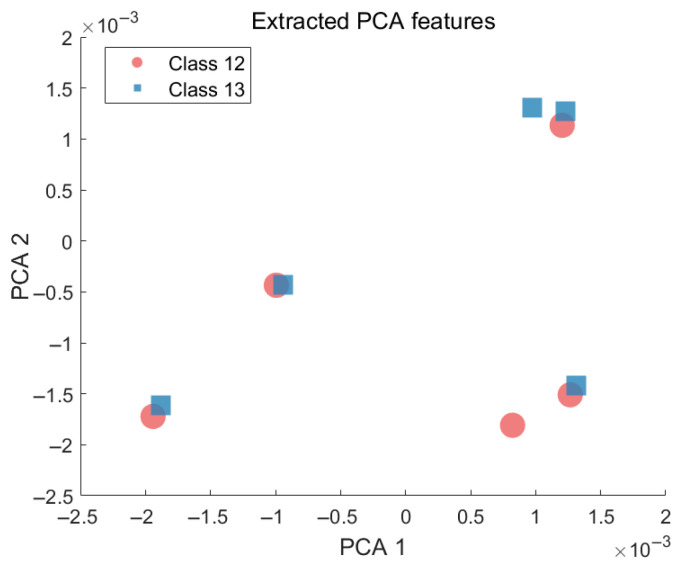
PCA-based projection of radiation source signal embeddings (Classes 12 and 13).

To complement the visual analysis, Principal Component Analysis (PCA) was further performed to quantify the spatial distribution of selected classes [27]. Figure 4 shows the PCA-based projection for Classes 12 and 13, and the corresponding feature coordinates are listed in Table 1. The results demonstrate overlapping feature regions and poorly defined boundaries, reinforcing the observations obtained from t-SNE.

In summary, these analyses indicate that the embedded feature space exhibits overlapping and dispersed structures, which pose significant challenges for traditional classification methods that rely on compact and well-separated class clusters. Detecting novel-class instances under such conditions requires more flexible mechanisms that do not assume closed-set distributions. This observation directly motivates the hybrid design adopted in the following section, which integrates deep feature extraction with unsupervised anomaly detection using iForest. The embedding characteristics revealed in this section are therefore directly aligned with the operational assumptions of the iForest module.

## 3. Proposed Method

**Figure 5:**
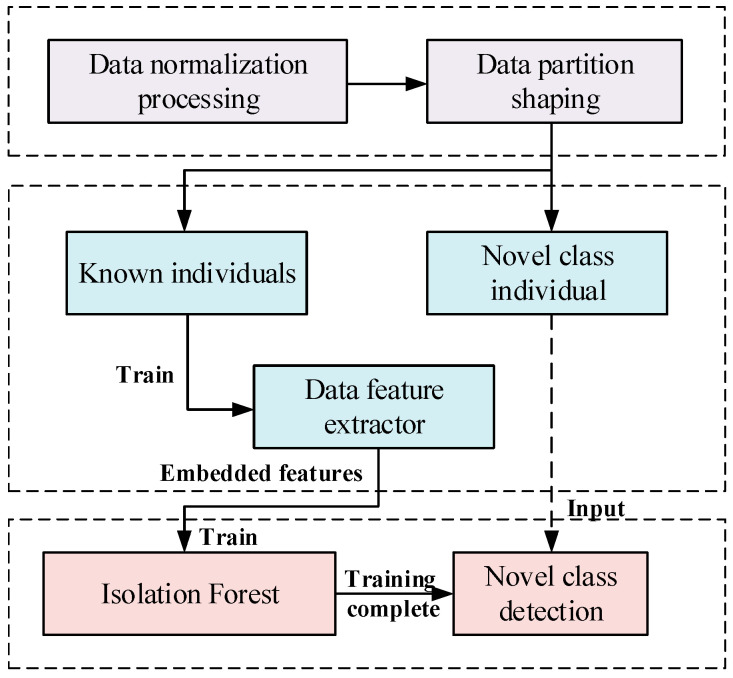
Overall flowchart of the proposed method.

We propose an IDFIF to address the limitations of existing radiation source recognition systems in detecting previously unseen individuals. Figure 5 illustrates the overall process of the proposed method.

The IDFIF method begins with data normalization and partitioning. Input signals are first preprocessed and then separated into two subsets: known-class samples and novel-class samples. Only the known-class data is used during the training phase. These samples are passed through a CNN-based feature extractor, which is optimized to produce compact and discriminative embeddings. This deep representation improves intra-class compactness and inter-class separability. The extracted embeddings are then used to train an iForest, which serves as an unsupervised anomaly detector. After training is complete, the iForest can evaluate test samples, including those from an unknown class. During inference, novel-class input signals are processed by the same feature extractor, and the resulting embeddings are passed to the iForest. Samples that deviate significantly from the distribution of known classes are assigned high anomaly scores and identified as novel-class candidates.

This mechanism builds upon the feature distribution patterns observed in Section 2, particularly the sparsity and overlap challenges within the embedding space. The iForest computes anomaly scores based on isolation depth across multiple randomly constructed trees, enabling effective separation between inliers and outliers without requiring prior knowledge of novel classes. By integrating deep representation learning with lightweight unsupervised detection, the IDFIF method achieves accurate, scalable, and data-efficient novel-class recognition in open electromagnetic environments.

### 3.1. Deep Feature Representation for Emitter Signal Embedding

To effectively capture the fine-grained modulation differences between individual radiation sources, we construct a CNN as the feature extractor to derive discriminative deep embedding features from preprocessed IQ signals. CNNs are particularly appropriate for this task because they can automatically learn structural patterns and remain robust to signal variations, which is essential given the observed intra-class diversity and inter-class overlap in ADS-B signals.

The feature extractor consists of multiple convolutional, pooling, and fully connected layers. It is trained with a Softmax classifier to promote compact intra-class clustering and improved inter-class separability in the learned feature space. The resulting embeddings provide stable and informative representations, which serve as high-quality input to the following novel class rejection module. The detailed network configuration is provided in Table 2, where *I* represents the number of known classes. No dropout or batch normalization layers are used in this configuration, and all convolutional layers employ ReLU activation and padding set to “same”. This architecture prioritizes simplicity while maintaining sufficient representational capacity for SEI tasks.

The loss function used by the feature extractor is formulated as follows:

(1) $$L_{\mathrm{fe}}=-\sum_{k=0}^{C}y_{k}\log(f(z_{k}))$$

where $y_{k}$ represents the true value of the sample. The $f(z_{k})$ is the output of the feature extractor, and the calculation formula can be expressed as:

(2) $$f(z_{k})=e^{z_{k}}/\left(\sum_{s}e^{z_{s}}\right)$$

where $e^{z_{k}}$ denotes the *k*-th output value of the fully connected layer. To evaluate the accuracy of the proposed model, we compute it using the following formula:

(3) $$\mathrm{acc}\_train=\frac{\delta_{t}}{\delta }$$

(4) $$\mathrm{acc}\_test=\frac{\delta_{t}^{\prime }}{\delta^{\prime }}$$

where *δ* is the number of all training samples, $\delta_{t}$ is the number of samples whose classification results are consistent with the labels during the training process, $\delta^{\prime }$ is the number of all testing samples, is the number of samples whose classification results are consistent with the labels during the testing process.

### 3.2. Novel Class Detection Using iForest

**Figure 6:**
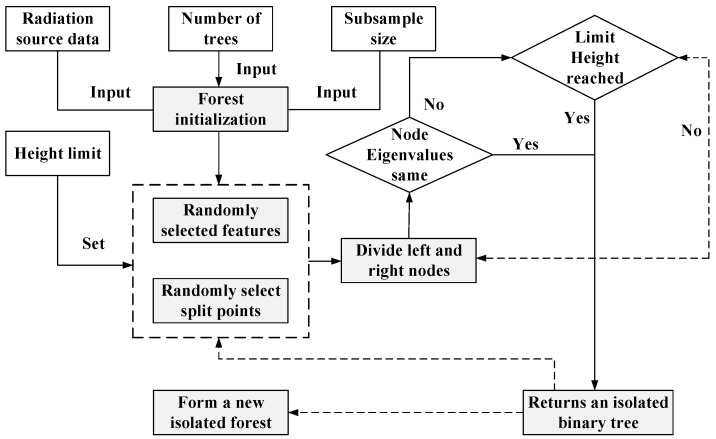
Process diagram for establishing a detection forest model based on iForest.

The proposed detection model is based on iForest, which detects novel-class samples by measuring how easily they can be isolated from known-class data in the learned embedding space. Specifically, the feature input to iForest is the vector from the final fully connected layer of the CNN, located immediately before the softmax activation. This embedding captures discriminative structures in the signal without introducing bias from classification probabilities. Such representations have proven suitable for unsupervised boundary modeling. The overall detection process is shown in Figure 6, which includes both training and evaluation stages.

In the training stage, the construction of the isolation tree is completed by recursively and randomly splitting the subsamples of the training set. This process continues until all samples are isolated or the preset maximum height is reached. It is necessary to set the value of the maximum height of the binary tree related to the total number of subsamples, which is roughly equal to the average height of the tree. During the growth process of the tree, if the average height is reached, the growth will stop. The reason is that the algorithm only needs to focus on those points with extremely short paths to determine whether the corresponding data belongs to novel-class individuals. Therefore, points with longer paths are not within the scope of attention.

The new individual detection model for radiation sources is mainly composed of multiple isolated binary trees, and constructing isolated binary trees is also the core of the entire method. The main construction steps are as follows:

(1) Randomly select $m_{s}$ sample points from the dataset D to form the required sample set $D_{S}$.
(2) Randomly select a feature $f_{iF}$ and a segmentation value $p_{iF}$ from the sample set $D_{S}$. If the maximum and minimum values of all samples contained in the node H on the feature $f_{iF}$ are fmax and *f_min_*, respectively, then $p_{iF}$ should be randomly selected from the interval $\left[f_{min},f_{max}\right]$.
(3) For each sample in the sample set, if the value of its feature $f_{iF}$ is less than the segmentation value $p_{iF}$, then the sample is assigned to the left child node of node H; If it is greater than or equal to $p_{iF}$, then it is assigned to the right child node of the node.
(4) Recursively repeat steps (2) and (3) for the left and right child nodes of node H to generate an isolated binary tree. The process stops until the number of samples in the child nodes is insufficient, there is only a single sample, or the height of the tree reaches the preset maximum value, that is, any one of the following conditions is satisfied: the tree reaches the limited height; there is only one sample in the node; all the feature values of the samples in the node are the same.
(5) Set a certain number of isolated binary trees and construct a detection model. For sample point $d_{j}$, obtain the corresponding path length $l(d_{j})$ in each isolated binary tree, and use a specific formula to calculate the new class determination score $S(d_{j},q)$ for $d_{j}$, in order to evaluate its likelihood of belonging to a new class individual.

The calculation formula for the new individual judgment score is as follows:

(5) $$S(d_{j},q)=2^{\frac{-E(l(d_{j}))}{c(q)}}$$

In Equation (5), q represents the total number of sample points for the new class model input by the user, $E(l(d_{j}))$ represents the average value calculated for all path lengths $l(d_{j})$, c(q) is the normalization of the height of the tree. Among them, the expression for c(q) is as follows:

(6) c(q)=2H(q−1)−2(q−1)/q

(7) H(w)=lnw+ξ

In the training stage, multiple isolated binary trees are constructed to establish a novel-class detection model. In the testing stage, the expected path length is calculated to obtain the novel-class score corresponding to each test sample. This score reflects the path length of the sample corresponding to each tree in the detection model. According to the calculated score, it can be determined whether the tested radiation source sample is a novel-class individual of the radiation source.

Calculate the determination score $S(d_{j},q)$ according to step 5, that is, the score for judging whether the current instance is a novel-class individual. Given that the feature distribution of the radiation source individual data shows a highly overlapping characteristic, introduce a novel-class discrimination coefficient based on the determination score for novel-class individuals and design the discrimination condition for novel-class individuals to obtain the final novel-class detection result $R_{j}$. The specific discrimination formula is shown in Equation (8).

(8) $$S(d_{j},q)>S_{\max},S(d_{j},q)>S_{mean}+\rho (S(d_{j},q)-S_{\max}),\rho \in \left[0,1\right]$$

In the above equation, $S_{max}$ represents the highest score among all new category samples, $S_{mean}$ represents the average score of all new category samples, and *ρ* represents the designed discriminant coefficient. If the score of the new category sample meets the above conditions, $R_{j}$ is marked as 1, indicating that it is a sample of the new category; Otherwise, $R_{j}$ is marked as 0, indicating that it is a sample of the old category.

Based on the frequency $R_{T}$ with a result of “1” and the total number of times the detection model was tested $I_{T}$, the accuracy μ of the new class detection can be calculated.

(9) $$\mu =\frac{R_{T}}{I_{T}}$$

The IDFIF can model known and unknown individuals without the need for prior information and a large amount of data. By searching for unknown individuals with “sparsity”, it can accurately perceive novel-class targets of radiation sources, enabling the individual radiation source recognition system to have the ability to perceive and judge unknown targets.

## 4. Experiment Evaluation

### 4.1. Experiment Setup

To evaluate the effectiveness of the proposed novel-class detection method, a series of experiments is conducted on the ADS-B-based individual radiation source dataset. Specifically, classes 0 to 38 (a total of 39 known classes) are selected as known-class samples to construct the training dataset. Additionally, several samples are randomly selected from class 39 as the “unknown class” to simulate novel-class individual radiation sources. These are used for the novel-class detection task in the testing stage. In this setting, the detection model is constructed using data from 40 distinct classes, but only the first 39 are exposed during training.

For the deep features obtained through the CNN-based embedding in Section 3.1, we adopt a randomized shuffling and splitting strategy to construct the training and testing sets. The known-class set comprises 2445 individual signals from the 39 known classes. To simulate novel-class detection tasks, samples from eight different internal subsets of class 39 are treated as novel-class instances, resulting in Experiments 1–8. To further evaluate the model’s ability to reject only truly novel classes without misclassifying known ones, Experiments 9–14 are constructed using randomly selected samples from six known classes.

During the training, the iForest was used to model the embedded features. Each time, training samples are proportionally (20%) from the 39 classes of old-class samples, and a total of 12,225 samples are sampled for training. In the testing stage, a total of 1000 detection simulations are carried out. The number of isolation trees is set to 8, and *ρ* is 0.67. The entire experiment is completed under the Windows operating system. The hardware environment includes a 13th Gen Intel(R) Core(TM) i5-13500H CPU and an NVIDIA GeForce RTX 4050 GPU. MATLAB 2022b is used for signal processing. The training of the feature extractor relies on the GPU environment, and the Adam optimizer is adopted. The specific training parameter settings are shown in Table 3.

To further verify the robustness and superiority of the proposed model, this paper selects six common detection and classification methods for NCSD task, including both classical and deep learning-based methods. The classical methods consist of Support Vector Machine (SVM), K-means clustering, and the K-Nearest Neighbors (KNN). The SVM is a supervised learning model based on hyperplane division, aiming to find an optimal decision boundary that maximizes the margin between different classes in high-dimensional space. K-means is an unsupervised clustering method that minimizes the intra-cluster variance to partition data into distinct groups. KNN is a non-parametric method that determines the class of a test sample based on majority voting among its K closest training neighbors in the feature space. These three approaches represent typical detection paradigms based on discriminative boundaries, clustering structure, and similarity measurement.

In addition, we further introduce three common used deep learning-based detection methods: Maxium Softmax Probability (MSP) [28], DeepSVDD [29], and OpenMax [21]. MSP is a confidence-based method that classifies a sample as novel if its predicted softmax probability is below a defined threshold. DeepSVDD is a one-class classification method that learns a compact hypersphere around known-class representations in the embedding space, treating samples that fall far outside this region as anomalies. OpenMax extends the traditional softmax layer by fitting Weibull distributions to activation vectors, thereby estimating the likelihood that a sample belongs to an unknown class.

These six methods provide a broad comparative framework encompassing supervised learning, unsupervised clustering, distance-based classification, confidence thresholding, and deep anomaly detection. Their inclusion enables a comprehensive evaluation of the proposed IDFIF method.

### 4.2. Evaluation Results

#### 4.2.1. Detection Performance of Novel-Class

**Figure 7:**
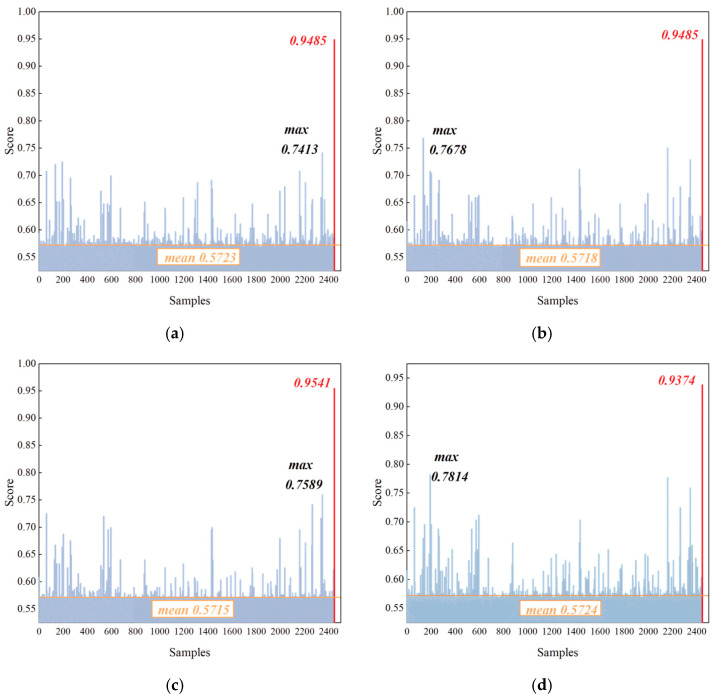

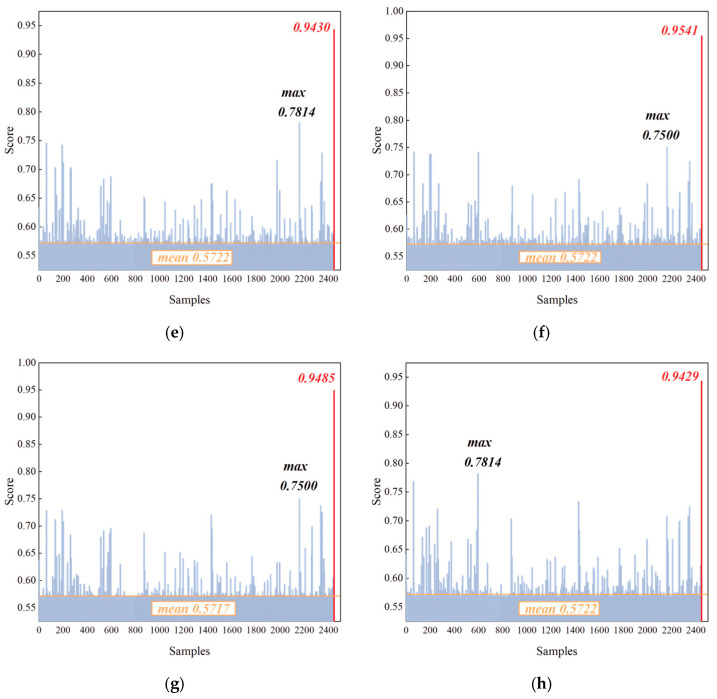
Anomaly score distributions for individual novel-class instances (**a**) score of experiment 1; (**b**) score of experiment 2; (**c**) score of experiment 3; (**d**) score of experiment 4; (**e**) score of experiment 5; (**f**) score of experiment 6; (**g**) score of experiment 7; (**h**) score of experiment 8.

To evaluate the detection capability of the proposed IDFIF method for novel-class instances, this section analyzes the results of Experiments 1–8. These experiments are designed to assess whether the method can effectively identify emitter signals that do not belong to any of the known training categories. The anomaly score distributions for eight randomly selected novel-class emitters are presented in Figure 7. Each subfigure contains 2446 samples, where the first 2445 represent known-class instances, and the final sample, which is highlighted by a red marker, corresponds to the injected novel-class instance.

As shown in Figure 7, the horizontal axis represents the index of test samples, and the vertical axis denotes the anomaly score assigned by the iForest model. The last sample in each sequence, marked in red, corresponds to the novel-class instance. All novel classes consistently exhibit significantly higher anomaly scores than those of the known-class samples, with values exceeding 0.9 in every case. This result demonstrates a clear separation in the learned feature space between known and novel classes, validating the effectiveness of the iForest in distinguishing outliers from the known-class distribution. Although iForest is originally designed for structured, low-dimensional feature spaces, the embedding generated by the CNN’s final hidden layer preserves localized class clusters and boundary structures that remain compatible with tree-based modeling. The stable anomaly scores shown in Figure 7 empirically support the suitability of this representation. The clear separation observed across all eight experiments indicates that random partitioning in this space can still yield consistent and meaningful detection boundaries. Furthermore, quantitative performance is summarized in Table 4. The detection accuracy for each experiment is computed according to Equations (7) and (8). All eight experiments achieved detection accuracies above 90 percent, with a mean accuracy of 94.83 percent and a variance of 0.000577. These results confirm both the accuracy and the stability of the IDFIF method in handling novel-class detection tasks.

In addition to detection accuracy, the IDFIF exhibits computational efficiency suitable for real-time applications. The CNN-based feature extractor performs a single forward pass per sample, and the iForest scoring process requires only *O*(*T log n*) operations over lightweight decision trees. As both modules are model-free at inference and support parallel processing, the overall pipeline remains practical for low-latency or embedded signal environments.

The superior performance of IDFIF is attributed to three core design advantages. First, the CNN-based feature extractor effectively compresses intra-class variability and enhances inter-class separability, which improves the quality of the embedding space. Second, the iForest functions as a robust unsupervised anomaly detector by modeling only the distribution of known-class data, which allows the detection of novel instances without prior exposure to unknown categories. Third, the hybrid design enables efficient open-set recognition in dynamic electromagnetic environments and reduces reliance on labeled data.

#### 4.2.2. False Positive Analysis for Known-Class Instances

**Figure 8:**
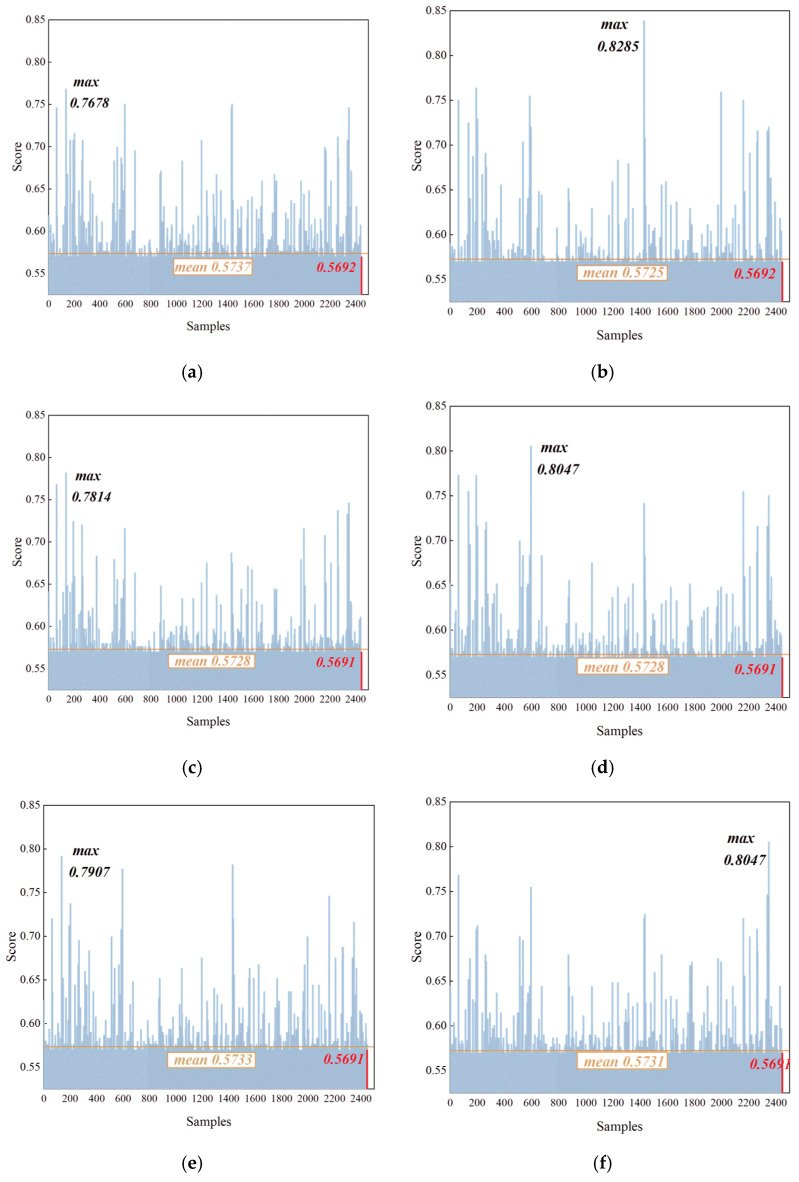
Anomaly score distributions for individual known-class instances (**a**) score of experiment 9; (**b**) score of experiment 10; (**c**) score of experiment 11; (**d**) score of experiment 12; (**e**) score of experiment 13; (**f**) score of experiment 14.

To evaluate the low sensitivity of the IDFIF method to known-category instances, specifically its rejection precision in novel-class detection with respect to minimizing false positives, this section analyzes the results of Experiments 9–14. The anomaly score distributions for six different known-class emitters are presented in Figure 8, and the corresponding results are summarized in Table 5. Each figure contains 2446 score values. The final sample, which is highlighted in red, represents the known-class instance artificially injected into the test set.

As shown in Figure 8, none of the known-class samples produced prominent anomaly scores. All final scores remained below the decision boundary and did not trigger the novel-class detection criterion. This result indicates that known-class instances, even those not seen during training, are not mistakenly identified as novel instances. The IDFIF method, therefore, demonstrates strong rejection precision. The classification results for these six experiments, computed according to Equations (7) and (8), are provided in Table 5. In all cases, the novel-class detection accuracy is recorded as zero, meaning that no known-class sample was misclassified as a novel instance. This empirical evidence supports the conclusion that the IDFIF method not only achieves high detection performance for novel classes but also exhibits excellent low sensitivity to known-class instances, which effectively reduces the risk of overgeneralization.

The robustness of this performance is attributed to the structure of the iForest-based detection mechanism. By modeling the global feature distribution of known classes, the iForest constructs anomaly boundaries that accurately delineate the limits of the training data space. As a result, it avoids misclassifying normally fluctuating known-class samples as outliers, ensuring stable and reliable detection performance in open-set environments.

#### 4.2.3. Comparative Experiments

This section presents a comparative analysis against six representative methods: SVM, K-means, KNN, MSP (Maximum Softmax Probability), DeepSVDD and OpenMax, to further evaluate the proposed IDFIF method’s relative performance in novel-class detection tasks. These methods represent a diverse range of detection paradigms, including distance-based classification, clustering, and deep anomaly detection. All comparative methods were evaluated independently using feature vectors extracted from the same CNN model used in the IDFIF. No integration or combination with the proposed method was involved.

**Figure 9:**
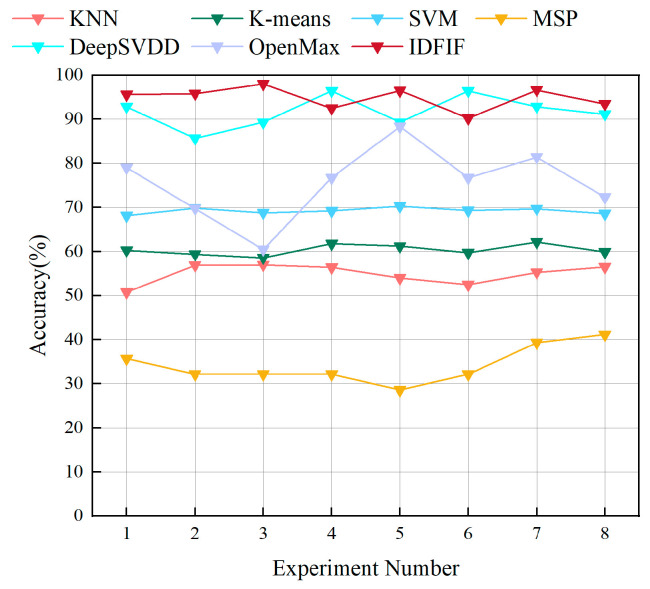
Comparison of detection accuracy for novel-class instances across different methods.

The experimental settings are consistent with those described in Section 4.2.1, where novel-class instances from Experiments 1 to 8 are used as the test set. The detection accuracy for each method is calculated accordingly, and the results are summarized in Figure 9 and Table 6.

As shown in Figure 9, the three classical methods exhibit limited performance. The KNN exhibits the lowest detection accuracy, consistently falling below 60%, indicating that its distance-based metric is insufficiently sensitive to anomalous signals in complex feature spaces. K-means shows slightly improved results, maintaining detection accuracy in the range of 59% to 62%, but still fails to deliver reliable detection. SVM achieves better performance, with accuracy rates near 70%, benefiting from hyperplane-based discrimination. However, as a supervised method, SVM relies heavily on the decision boundaries established during training and tends to exhibit limited generalization when encountering novel classes. Among deep learning-based methods, MSP performs worst, with accuracy ranging between 28.6% and 41.1%, confirming that softmax confidence is not a reliable novelty signal., OpenMax improves over MSP, achieving moderate results (around 60–80%), but with noticeable variance across experiments. DeepSVDD exhibits strong performance, achieving accuracy above 90% in most cases, validating the strength of one-class deep modeling in novel-class detection tasks.

In contrast, the proposed IDFIF method consistently achieves the highest accuracy across all eight experiments, never dropping below 90%. Its average accuracy exceeds 94%, and in Experiment 3, it reaches 98%. Compared to other methods, IDFIF provides stable and superior performance, highlighting its robust integration of discriminative feature learning and unsupervised anomaly detection.

These results clearly demonstrate that the IDFIF method outperforms both classical and deep learning-based methods in novel-class detection tasks. Its superior performance highlights the robustness of the learned feature representations and the effectiveness of the unsupervised boundary modeling strategy. These findings indicate that IDFIF provides strong generalizable and scalability for practical signal intelligence applications.

In summary, the comparative experiments further validate the advantages of the proposed IDFIF method in open-set scenarios. By integrating deeply embedded feature representation with unsupervised modeling, IDFIF eliminates the reliance on prior knowledge of novel classes and consistently achieves high accuracy and stability. These results confirm its potential as a practical and scalable solution for enhancing novel-class awareness in radiation source identification systems.

## 5. Conclusions

This paper addresses the challenge faced by radiation source identification systems in complex electromagnetic environments when encountering previously unseen emitter classes. To overcome this challenge, a novel-class detection model, termed IDFIF, is proposed by integrating deep feature representation with an iForest-based decision mechanism. The method employs a CNN to extract discriminative features from emitter signals, which effectively mitigates the classification difficulties caused by large intra-class variation and small inter-class differences. Furthermore, modeling the boundary of known-class feature distributions using the iForest method is capable of accurately detecting and rejecting novel-class emitter instances in an unsupervised manner, without requiring any prior knowledge of new classes.

Experimental results demonstrate that the proposed method significantly outperforms classical approaches such as SVM, K-means and K-NN in terms of novel-class detection accuracy, achieving an average accuracy exceeding 94%. In addition, the method exhibits strong low sensitivity to known-class samples, which effectively prevents false rejections. Even in scenarios with blurred inter-class boundaries or severe intra-class feature drift, the method maintains stable discriminative performance, indicating excellent generalization and robustness.

In conclusion, the IDFIF model not only enhances the open-set awareness capability of radiation source identification systems but also provides a practical and scalable technical pathway for constructing SEI frameworks with open-set recognition capabilities. While current evaluations are conducted under stable channel conditions, the method shows strong potential for generalization to more dynamic or adverse electromagnetic environments. To rigorously evaluate this potential, future work will explore adaptive thresholding strategies to improve responsiveness under varying SNR conditions and channel impairments. Moreover, although the current framework detects novel classes on a per-sample basis, it remains applicable in situations where multiple unknown emitters appear simultaneously. Enabling more advanced joint modeling of multiple novel classes under unsupervised settings is considered a promising direction for future work, especially in dense signal environments. In addition, integrating meta-learning and incremental learning could enable continuous adaptation to novel knowledge across multiple scenarios and protocols. Moreover, although this study is based on ADS-B signals, the proposed method is generalizable to other wireless signal types such as Wi-Fi, LoRa, and ZigBee, provided that the signal characteristics allow for stable feature embedding. This extensibility highlights the method’s broader applicability across RF domains. These research directions will further promote the development of next-generation SEI systems with greater intelligence, real-time capability, and adaptability.

## Figures and Tables

**Table 1:** PCA coordinates of radiation source signals for Classes 12 and 13.

Category	Class 12				
Sample	1	2	3	4	5
PCA1	−9.94 × 10^−4^	8.23 × 10^−4^	12.66 × 10^−4^	−19.41 × 10^−4^	12.04 × 10^−4^
PCA2	−4.35 × 10^−4^	−18.09 × 10^−4^	−15.10 × 10^−4^	−17.22 × 10^−4^	11.34 × 10^−4^
**Category**	**Class 13**				
**Sample**	**6**	**7**	**8**	**9**	**10**
PCA1	9.75 × 10^−4^	−9.39 × 10^−4^	13.13 × 10^−4^	−18.81 × 10^−4^	12.31 × 10^−4^
PCA2	13.10 × 10^−4^	−4.35 × 10^−4^	−14.17 × 10^−4^	−16.10 × 10^−4^	12.74 × 10^−4^

**Table 2:** Main structural parameters of the feature extractor.

Layer Type	Parameters	Activation
Input	3000 × 2 × 1	
Conv2d	Kernel:20 × 1, Filters: 20, Stride:1	ReLU
Max pool2d	Pool size: 2 × 1, Stride:2 × 1	
Conv2d	Kernel:20 × 1, Filters: 40, Stride:1	ReLU
Max pool2d	Pool size: 2 × 1, Stride:2 × 1	
Conv2d	Kernel:20 × 1, Filters: 60, Stride:1	ReLU
Max pool2d	Pool size: 2 × 1, Stride:2 × 1	
Fullyconnected	20	
Fullyconnected	*I*	
Softmax		

**Table 3:** Feature extractor training parameters.

Parameters	Value
Training environment	GPU
MaxEpochs	30
MiniBatchSize	128
Initial learning rate	0.001
Gradient threshold	1
Optimization algorithm	Adam

**Table 4:** Detection accuracy of the IDFIF method on novel-class emitter instances.

Experiments	1	2	3	4	5	6	7	8
Accuracy (%)	95.6	95.8	98.0	92.5	96.5	90.2	96.6	93.5

**Table 5:** Detection accuracy of the IDFIF method on known-class emitter instances.

Experiments	9	10	11	12	13	14
Accuracy (%)	0	0	0	0	0	0

**Table 6:** Detection accuracy of the IDFIF method and baseline methods on novel-class instances.

Experiments	1	2	3	4	5	6	7	8
KNN	50.8	56.9	57.0	56.4	54.0	52.5	55.3	56.5
K-means	60.3	59.4	58.6	61.8	61.2	59.7	62.1	59.9
SVM	68.1	69.8	68.8	69.2	70.3	69.3	69.7	68.6
MSP	35.7	32.1	32.1	32.1	28.6	32.1	39.3	41.1
DeepSVDD	93.0	85.7	89.3	96.4	89.3	96.4	92.8	91.1
OpenMax	79.1	69.8	60.5	76.7	88.4	76.8	81.4	72.3
IDFIF	95.6	95.8	98.0	92.5	96.5	90.2	96.6	93.5

## Data Availability

Data are contained within the article.
